# Exploring parents’ perceptions and experiences of childhood obesity and management in Riyadh, Saudi Arabia: an interpretive qualitative study

**DOI:** 10.1186/s12889-024-21014-6

**Published:** 2024-12-18

**Authors:** Sarah Hamad Almutairi, Sami Abdulrahman Alhamidi

**Affiliations:** 1 College of Nursing, King Saud University, Riyadh, 12372 Saudi Arabia; 2 Department of Psychiatric & Mental Health & Community Health Nursing, College of Nursing, Qassim University, Qassim, 52571 Saudi Arabia; 3 Department of Maternal and Child Health, College of Nursing, King Saud University, Riyadh, 12372 Saudi Arabia

**Keywords:** Childhood obesity, Parental perceptions, Saudi Arabia, Qualitative study, Hybrid thematic analysis, Public health intervention

## Abstract

**Background:**

Childhood obesity is a public health concern in Riyadh, Saudi Arabia, where cultural and social factors shape parental perceptions. This study explores how Riyadh-based parents view childhood obesity.

**Methods:**

A hybrid approach to thematic analysis was employed, combining deductive and inductive coding to allow for emergent themes directly from the data. Semistructured interviews were conducted with twelve parents in Riyadh, Saudi Arabia, whose children were identified as overweight or obese. The data were analysed to identify key themes related to parental perceptions and childhood obesity management.

**Results:**

Four themes emerged from the data: (1) perceptions towards childhood obesity; (2) perceived barriers to weight management; (3) perceived benefits to weight management; and (4) perceived motivators to addressing obesity.

**Conclusion:**

Parents in Riyadh often view childhood obesity as a sign of health due to deep-rooted cultural norms. However, as they witness health and social challenges in their children, such as bullying or physical difficulties, their perceptions begin to shift. This study highlights the role of cultural beliefs, lifestyle constraints, and limited institutional support as barriers to managing childhood obesity. Addressing these factors through awareness initiatives and community support could empower parents to more effectively promote healthier behaviours for their children, ultimately contributing to improved health outcomes.

**Supplementary Information:**

The online version contains supplementary material available at 10.1186/s12889-024-21014-6.

## Background

Childhood obesity is a growing global public health issue, with over 390 million children and adolescents aged 5–19 years overweight and 160 million living with obesity, according to the World Health Organization’s 2022 report [1]. This trend poses significant long-term health risks, including cardiovascular diseases, type 2 diabetes, and psychological issues, which often extend into adulthood [2]. Contributing factors such as poor diet, sedentary lifestyles, and changing socioeconomic conditions [3] make childhood obesity a complex problem requiring coordinated global action to prevent and mitigate its impact.

In Saudi Arabia, the rising rates of childhood obesity are especially alarming. A recent study indicated that approximately 11.2% of Saudi children and adolescents are either overweight or obese, with the highest prevalence among boys aged 2–6 years [4]. Furthermore, a recent study in Riyadh highlighted significant health concerns among children, finding an obesity prevalence rate of 18.2% [5]. Another 2022 study in Saudi Arabia’s Eastern Province, involving 20,000 children, reported even higher figures, with overweight and obesity rates among high school students reaching 25.7% [6]. Studies from Saudi Arabia confirm that obesity during childhood is a key predictor of obesity in adulthood [7], which is associated with a multitude of adverse health outcomes, including cardiovascular diseases [8], type 2 diabetes [9], and psychological disorders, including depression and anxiety [10].

Multiple factors contribute to the high rates of childhood obesity in Saudi Arabia, and these factors are often influenced by a combination of sociocultural [11], socioeconomic [5], and behavioural factors [12]. Rapid urbanisation and economic growth in the country have prompted significant dietary shifts towards energy-dense, high-calorie foods, mirroring Western dietary patterns [13]. Low levels of physical activity, compounded by sedentary behaviours and cultural barriers, significantly contribute to the growing childhood obesity problem in Saudi Arabia, with many children and adults leading predominantly inactive lifestyles [14]. However, owing to the sociocultural cohesion of the Saudi community, it is believed that parents play a significant role in influencing childhood obesity in Saudi Arabia.

The role of parents in managing childhood obesity is critical, as their perceptions of their children’s weight status significantly influence their attitudes and behaviours towards managing and preventing obesity. However, many Saudi parents fail to accurately perceive their children’s weight, often underestimating or misjudging their child’s obesity status [15]. This misperception can hinder early intervention and exacerbate the problem of childhood obesity. Furthermore, parental educational levels and socioeconomic status are strongly linked to childhood obesity [5]. Cultural beliefs also play a significant role in shaping parental perceptions of childhood obesity. In Saudi society, a ‘larger body size’ is often linked with health and affluence, which results in parents perceiving overweight children as healthy [15]. This cultural perception and social norms can pose challenges to efforts aimed at promoting healthier lifestyles and preventing childhood obesity in Saudi Arabia [16]. However, there is a limited understanding of how these cultural beliefs specifically shape parental perceptions of childhood obesity among Saudi Arabian children in Riyadh, highlighting a critical research gap.

Despite the increasing awareness of childhood obesity in Saudi Arabia, there is a need for culturally tailored interventions that address parental perceptions and behaviours. Previous studies have highlighted the need for educational programmes to improve parental awareness of childhood obesity and its associated health risks, with a focus on encouraging healthy eating habits and increasing physical activity among children [17–19]. For example, school-based initiatives and national programmes, such as the RASHAKA programme, have made strides towards addressing childhood obesity by promoting healthy diets and physical activity; however, further efforts are required to sustain and expand these initiatives [20].

In light of the complex sociocultural, socioeconomic, and behavioural factors influencing parents’ perceptions of childhood obesity in Riyadh, Saudi Arabia, this study aims to explore these perceptions and the underlying factors that shape them. By understanding parental beliefs and attitudes, it is possible to inform more effective intervention strategies that are culturally relevant and sustainable.

## Methods

### Study design

This study utilised a qualitative approach with an interpretive philosophical paradigm to explore Saudi parents’ perceptions and experiences regarding childhood obesity. The interpretive qualitative approach was chosen because it allows for a naturalistic and in-depth exploration of participants’ subjective experiences [21]. This approach aligns with the study’s aim to understand how parents in Riyadh, Saudi Arabia, perceive childhood obesity.

### Participants and sampling

A purposive sampling technique was employed to recruit participants for this study [22]. Participants were selected based on having children who were classified as overweight or obese. In qualitative research, purposive sampling is employed to access individuals who meet specific criteria based on their experiences, knowledge, or distinct characteristics, enabling them to provide insights into the research question and thereby enhancing the depth and richness of the data collected [22]. The study included parents living in Riyadh, Saudi Arabia, with children aged 5 to 18 years who had a Body Mass Index (BMI) in the 85th percentile or higher. The exclusion criteria included parents of children with underlying medical conditions contributing to obesity or those whose children did not meet the overweight or obese BMI criteria. Riyadh, the capital of Saudi Arabia, is a large urban area and a major metropolitan hub, reflecting diverse socio-economic backgrounds and lifestyles that influence health-related behaviours.

Participants were initially approached through obesity support groups on social media platforms (WhatsApp and Telegram), where information about the study and eligibility criteria were shared. These two social media platforms were selected due to their widespread use among the target population and their effectiveness in reaching diverse demographic groups within Riyadh. A standardised message, including an invitation to participate and study details, was shared via posts and direct messages. Those interested in participating contacted the research team directly for screening to confirm they met the study’s inclusion criteria. Snowball sampling was used alongside purposive sampling, where enrolled participants were encouraged to refer other eligible parents within their network, thus broadening the reach to include a diverse sample within Riyadh. Out of 19 parents contacted through social media platforms, 12 agreed to participate, while seven parents declined or did not respond. Finally, a total of 12 participants were interviewed. Data saturation was achieved by the 10th interview, but all scheduled participants were interviewed for confirmation.

### Data collection

Data were collected through semistructured interviews conducted via Zoom, ensuring flexibility and convenience for participants. All participants confirmed that they resided in Riyadh at the time of the study. Although interviews were conducted via Zoom, recruitment was targeted within Riyadh to ensure that participants reflected the cultural and social context specific to this urban environment. The research team developed a semistructured interview guide, which was subsequently reviewed by three experts experienced in public health research, and then piloted with two volunteers. The interview guide included a range of questions, with examples highlighted in supplementary file 1.

This study was guided by the Health Belief Model (HBM), which provided a theoretical framework for developing the interview guide and interpreting findings. Originally developed by social psychologists to predict health-related behaviours, the HBM incorporates constructs such as perceived susceptibility, severity, benefits, and barriers, along with cues to action and self-efficacy [23]. These constructs were used to shape interview questions exploring parents’ perceptions, motivations, and challenges in managing childhood obesity, allowing for a structured yet flexible approach to understanding parental beliefs within the Saudi context.

Each interview began with an open-ended question to encourage participants to share their experiences in their own words. For instance, the first question in the interview guide was broad: ‘*Could you please tell me about your experience with your child’s obesity?*’ This approach facilitated a natural flow of conversation, enabling rich, detailed responses through a flexible interview guide that allowed for follow-up questions, prompts, and additional commentary [24]. The interviews were conducted over a period of four months, from March 2024 to June 2024. This timeline allowed for thorough recruitment and data saturation, ensuring a comprehensive range of parental perspectives on childhood obesity in Riyadh. Each interview lasted between 30 and 40 min and was audio-recorded with the participant’s consent. The interviews were conducted in Arabic, and transcripts were subsequently translated into English using a back-translation method, following the guidelines outlined by Alzyood et al. (2020), to ensure accuracy [25]. Additionally, member checking was performed by sharing transcripts with participants for verification of their responses.

### Data analysis

Thematic analysis, utilising a hybrid approach of both inductive and deductive coding, was used to analyse the data [26]. This method was chosen because of its flexibility and capacity to capture both predefined themes and emergent ones from the data. The hybrid thematic analysis was conducted in three phases following Swain’s (2018) model, ensuring a systematic and rigorous process of coding and theme development. The researcher (S.H.A) identified and coded both a priori themes on the basis of the study’s objectives and theoretical framework and a posteriori themes emerging from the data. Data was managed using the NVivo© 14 software with highlight and table functions created to summarise and organise codes across all transcripts. In the thematic analysis, the second researcher (S.A.A) independently reviewed and confirmed the initial codes, themes, and subthemes. This independent analysis was followed by discussions among all researchers (S.H.A, S.A.A), leading to mutual agreement on the final coding framework and thematic structure, reducing single-researcher bias, and ensuring the robustness and reliability of the findings [27].

### Ethical considerations

The study was approved by the King Saud University Human Research Ethics Committee (No. KSU-HE-24-043). Informed consent was obtained from all participants, and they were assured of their right to withdraw at any stage. Confidentiality and anonymity were maintained throughout the study by using pseudonyms and securely storing data on a password-protected computer. Only the researcher and supervisor had access to the raw data.

## Results

### Overview of participants

Twelve parents, consisting of ten females and two males, participated in the study. All participants were professionals (*n*=8), students (*n*=1), or housewives (*n*=3). The participants’ ages ranged from 28 to 46 years, and their children’s ages ranged from 5 to 15 years, with seven boys and five girls. The majority of the participants (nine out of twelve) had a university education, two had a primary level of education, and one had an intermediate level of education. The BMI values for the children in this study indicate that the majority of participants fall within the obese range (>95th percentile), with only a few falling in the overweight category (85th–95th percentile) (Table 1).

**Table 1:** Participants profiles

Participant Number	Parent & Living Arrangement	Age of the Parent	Level of Education & Profession of the Parent	Age & Gender of the Child	Child BMI & Range
1	Mother & parents live together	44	University level & Housewife	12 – Female	26.48 - >95% Obese
2	Mother & parents live together	37	University level & Nurse	11 – Male	24.49 - >95% Obese
3	Father & parents live together	34	University level & Manager	10 – Female	27.01 - >95% Obese
4	Mother & parents live together	28	University level & Student	5 – Male	22.68 - > 95% Obese
5	Father & parents live together	46	University level & Teacher	15 – Male	32.27 - >95% Obese
6	Mother & parents live together	40	University level & Teacher	13 – Male	30.70 - >95% Obese
7	Mother & parents live together	38	Intermediate level & Housewife	9 – Female	35.71 - >95% Obese
8	Mother & parents live together	35	University level & Nurse	9 – Male	34.96 - >95% Obese
9	Mother & parents live together	36	University level & Teacher	8 – Female	19.15–85- 95% Overweight
10	Mother & parents live together	41	Primary level - housewife	8 – Female	35.6 - >95% Obese
11	Mother & parents live together	32	Primary level - Employee	8 – Male	23.19 - >95% Obese
12	Mother & parents live together	35	University level & Teacher	10 – Male	21–85- 95% overweight

### Key themes

**Fig. 1:**
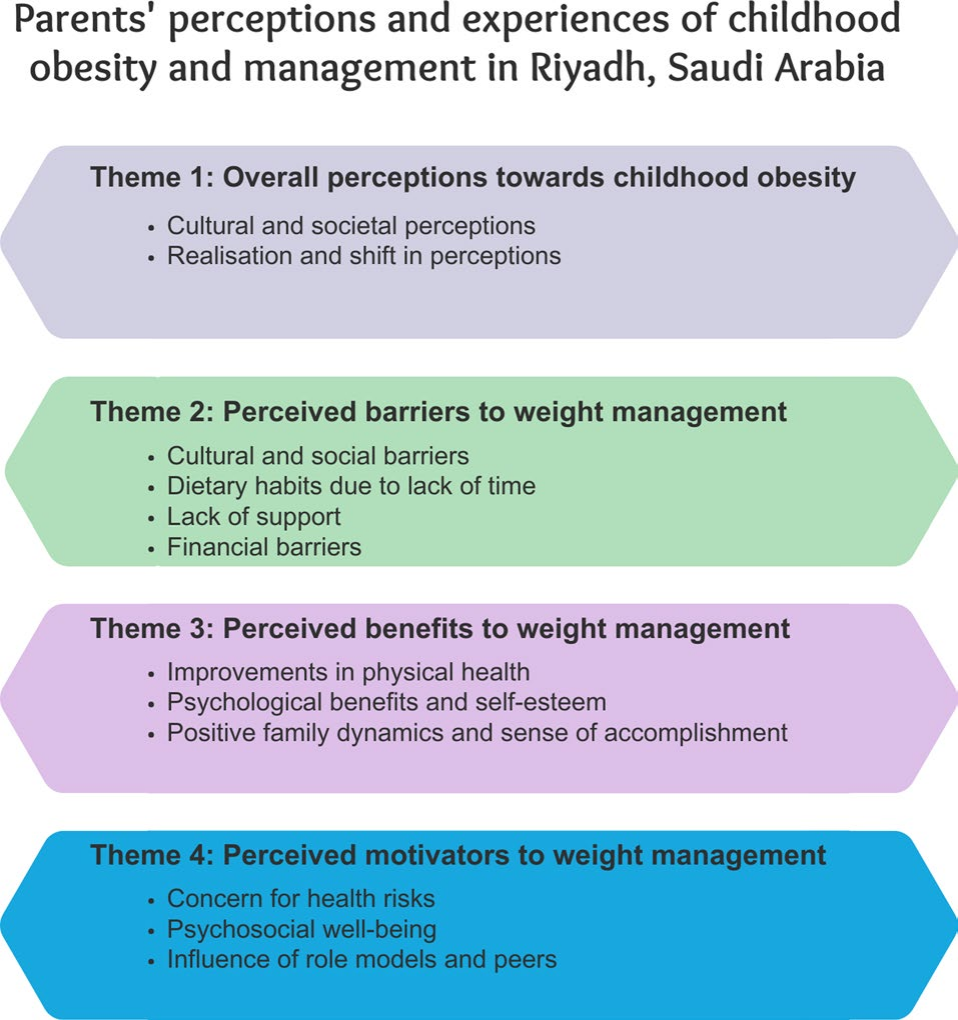
Thematic map, themes and subthemes

The thematic analysis of the interviews revealed four key themes reflecting the participants’ perceptions of childhood obesity and its management: (1) perceptions towards childhood obesity, (2) perceived barriers to weight management, (3) perceived benefits to weight management, and (4) perceived motivators to addressing obesity. These themes emerged from the analysis of 51 primary codes, which were derived from both deductive and inductive coding approaches (Fig. 1). Table 2 summarises these themes, their exemplar meaning unit and illustrative quotations from participants.

**Table 2:** Thematic analysis of parents’ perceptions, barriers, motivators, and benefits of childhood obesity management

Theme	Sub-themes	Exemplar Meaning Unit	Participant Number
**(1)** **Overall perceptions towards childhood obesity**	Cultural and societal perceptions	“It is always the older generation who encourage children to eat more as a sign of good appetite and overall health, they ask if the kid is ok if not eating a lot.” “In our culture [referring to the Saudi culture], we see a child who is chubby as healthy and well-fed. It’s like a sign that they’re being taken care of.”	Parent 7 Parent 3
	Realisation and shift in perceptions	“I realised the seriousness of obesity when my child faced health and social problems.” “I started to worry when she began to have health problems and struggled to move.”	Parent 2 Parent 4
**(2)** **Perceived barriers to weight management**	Cultural and social barriers	“The old beliefs that a chubby child is a healthy child are hard to change. Even when my son was overweight and had vitamin deficiencies, people still believed he was healthy because he was fat.”	Parent 11
	Dietary habits due to lack of time	“My girl mimics vloggers, YouTubers, and anybody who advertises a meal she wants to try.” “Because of my work schedule, I am often not at home to prepare meals. This means my child ends up eating a lot of fast food and snacks.”	Parent 1 Parent 12
	Lack of support	“There is no support from my family. In Saudi culture, family gatherings often involve rewarding children with restaurant meals or sweets.” “We never had the appropriate health education from health institutions.”	Parent 4 Parent 3
	Financial barriers	“Healthy things are not available to him all the time; they are expensive, such as flax seeds and brown bread, so we are forced to take unhealthy alternatives.” “I’ve thought about taking her to a club a few times. Even her mother says, ‘Why don’t you take her to a club?’ But sometimes my budget is a little tight.”	Parent 10 Parent 12
**(3) Perceived benefits to weight management**	Improvements in physical health	“After my son lost weight, he became more active. He could play and run around without getting tired as quickly, and that was a huge relief for me.” “My daughter had issues with her breathing and felt exhausted all the time. Since starting on a healthier diet and regular exercise, she breathes better and isn’t as lethargic.”	Parent 6 Parent 4
	Psychological benefits and self-esteem	“My child used to be so shy and withdrawn, but after losing weight, she has become more confident. She talks more and is happier to be around other kids.” “My son was bullied at school because of his weight. Since losing weight, he’s more comfortable with himself, and the bullying has stopped.”	Parent 2 Parent 3
	Positive family dynamics and sense of accomplishment	“When I started supporting my daughter in her weight loss journey, we grew closer. It wasn’t just about her losing weight; it was about spending quality time together and working towards a goal.” “It is not just about my child being physically healthier, but also seeing him more confident, happier, and enjoying life. That makes all the effort worth it.”	Parent 10 Parent 5
**(4) Perceived motivators to addressing obesity**	Concern for health risks	“The doctor said my child was at risk for diabetes, and I was scared; I knew we had to change our lifestyle.” “I want my daughter to grow up healthy, go to university, and not worry about her weight. If she learns healthy habits now, they will last for life.”	Parent 8 Parent 9
	Psychosocial well-being	“I don’t want my child to feel different or excluded because of his weight. It affects his happiness and confidence.” “It’s tough to see your child left out of games because they’re not as fit as other kids. I wanted to give him a chance to be included.”	Parent 10 Parent 11
	Influence of role models and peers	“I saw a child in our neighbourhood who lost a lot of weight. I thought, if he could do it, so can we.” “My sister’s son managed to lose weight, and seeing that transformation made me believe we could do the same for our child.”	Parent 6 Parent 7

### Theme 1: perceptions towards childhood obesity

The perceptions of childhood obesity among Saudi parents are shaped by cultural norms, familial beliefs, and awareness of health risks. This theme examines the evolving understanding of childhood obesity, revealing how traditional viewpoints often conflict with emerging health concerns. It is divided into two sub-themes: cultural and societal reinforcement of perceptions, and realisation and shift in perceptions.

#### Cultural and societal reinforcement of perceptions

Many parents initially viewed childhood obesity as a positive sign, equating it with good health and proper nourishment. Cultural beliefs strongly influenced these perceptions, as being ‘chubby’ was often associated with vitality and well-being. For instance, one parent remarked, ‘*The chubbier the girl and the more plump her cheeks*,*the healthier she is*’ (Parent, 2), highlighting the entrenched view that larger body size equates to better health. Another parent explained, ‘*I used to believe that obesity meant good health*’ (Parent, 4). These beliefs come from extended family or older generations, which further entrenches these cultural norms. One parent stated, “*It is always the older generation who encourage children to eat more as a sign of good appetite and overall health*,*they ask if the kid is ok if not eating a lot”* (Parent, 7). Such beliefs contributed to feeding practices where parents, aiming to promote what they perceived as health, inadvertently encouraged overconsumption and weight gain. A mother shared, ‘*I started receiving recommendations from family and neighbours*,*and you know*,*some families have the perception that the chubbier the child*,*the healthier they are. So*,*I began to hear this and started to believe that my child was too thin*,*so I began giving him foods that were supposed to increase his weight*’ (Parent, 10).

Parents’ perceptions of childhood obesity were shaped by cultural norms and reinforced by family and community settings, where physical traits like chubbiness were often viewed as indicators of health and vitality. In Saudi culture, a “chubby” child is frequently seen as a symbol of well-being and prosperity. For example, one parent noted, ‘*In our culture [referring to the Saudi culture]*,*we see a child who is chubby as healthy and well-fed. It’s like a sign that they’re being taken care of*.’ (Parent, 3).

Family interactions further reinforced these beliefs, with elders and relatives encouraging children to eat more and equating a healthy appetite with good health. One parent explained, ‘*In my family*,*it’s common to serve big portions to children*,*and if they don’t eat*,*people worry. They say*, ‘*Why isn’t your child eating well? Is he sick?’ It makes you think that a healthy appetite equals a healthy child*.’ (Parent, 5). Another parent shared how this cultural perception was celebrated in their community, ‘*When my daughter was a toddler*,*people would say how cute she was because she was chubby. It was seen as a good thing*,*a sign that she was well-cared for*.’ (Parent, 7).

Some parents began to question these beliefs as awareness of the health risks associated with obesity grew. However, they described challenges in shifting away from traditional practices. One parent recounted, ‘*I used to think that feeding my child until he was full – really full – was a good thing*,*but then I learned that it’s not always healthy. It’s hard to change the way we were brought up*,*but now I see the importance of balance*.’ (Parent, 8).

However, as parents began to notice health challenges in their children, this perception started to evolve.

#### Realisation and shift in perceptions

Over time, many parents reported a shift in their understanding of childhood obesity, often prompted by the physical challenges their children faced or by advice from healthcare professionals. Initially, many parents did not view obesity as a serious concern, but as they observed their children experiencing difficulties—such as trouble with movement, breathing problems, or social issues like bullying—they began to recognise the health risks associated with it. One parent explained, ‘*I realised the seriousness of obesity when my child faced health and social problems* (Parent, 2). Another parent shared, ‘*I started to worry when she began to have health problems and struggled to move*’ (Parent, 4).

As parents began to recognise the health risks associated with childhood obesity, they also identified several barriers to effectively managing their children’s weight. The following theme explores these perceived barriers in greater detail.

### Theme 2: perceived barriers to weight management

Participants identified several barriers to effectively managing their children’s weight. This theme explores four key sub-themes on the perceived barriers parents face in effective weight management: cultural and social barriers, dietary habits due to lack of time, lack of support, and financial barriers.

#### Cultural and social barriers

Societal beliefs equated a ‘chubby’ child with being healthy and prosperous. Another parent reflected on the challenges of changing these ingrained beliefs: ‘*The old beliefs that a chubby child is a healthy child are hard to change. Even when my son was overweight and had vitamin deficiencies*,*people still believed he was healthy because he was fat*’ (Parent, 11).

Additionally, social influences, including family and friends, often reinforced unhealthy eating habits. When parents attempted to make dietary changes, they were met with resistance or contradictory actions from their community: ‘*If I refused to give my child fizzy drinks when we met with others*,*they would contradict me and give him soda*,*chocolate chips*,*and such things*’ (Parent, 6). Societal pressure to conform to traditional norms around food consumption often undermined parents’ efforts to promote healthier lifestyles for their children.

#### Dietary habits due to lack of time

Parents acknowledged that their busy work schedules and the availability of fast food made it difficult to provide nutritious meals and maintain an active lifestyle for their children. Many relied on calorie-dense, nutrient-poor foods due to their convenience. A participant noted, ‘*Because of my work schedule*,*I am often not at home to prepare meals. This means my child ends up eating a lot of fast food and snacks*’ (Parent, 12). The lack of time for meal preparation and physical activities led to a reliance on processed foods, contributing to the children’s weight issues. A parent expressed frustration with the lack of time: ‘*The limited time and lack of sufficient knowledge about the correct diet*’ (Parent, 2). Similarly, another parent stated, ‘*My work schedule sometimes requires me to be out late*,*making it difficult to maintain a consistent*,*healthy meal plan at home’* (Parent 3).

In addition, children’s food preferences often leaned towards unhealthy options, which made it challenging for parents to introduce healthier alternatives. One mother shared how her child’s food preferences were influenced by social media: ‘*My girl mimics vloggers*,*YouTubers*,*and anybody who advertises a meal she wants to try*’ (Parent, 1).

#### Lack of support

Parents expressed frustration over the lack of consistent support from schools, healthcare providers, and family members in managing their children’s obesity. Many parents felt that schools could do more, such as offering healthier food options and promoting physical activity. One parent shared, ‘*There was no support from the school; I never received any message saying my daughter is suffering from obesity*’ (Parent, 4). In addition, parents highlighted the absence of clear health education from healthcare providers, which left them feeling unsupported in managing their children’s weight. As one parent noted, ‘*We never had the appropriate health education from health institutions*’ (Parent, 3).

Family members also played a role in undermining efforts to manage weight, with many parents reporting that family gatherings often involved rewarding children with unhealthy foods. One parent shared, ‘*There is no support from my family. In Saudi culture*,*family gatherings often involve rewarding children with restaurant meals or sweets’* (Parent, 7). Moreover, a lack of community resources, such as accessible parks or gyms, further limited opportunities for physical activity. ‘*I wish there were more accessible places like parks or gyms for children near our home’* (Parent, 9).

#### Financial barriers

For some families, financial constraints were a significant barrier to healthy eating and access to physical activities. Healthier food options were often more expensive and not as readily available. One parent shared, ‘*Healthy things are not available to him all the time; they are expensive*,*such as flax seeds and brown bread*,*so we are forced to take unhealthy alternatives*’ (Parent, 10). Another parent noted that while they wanted to enrol their child in a sports club for exercise, the cost was prohibitive: ‘*I’ve thought about taking her to a club a few times. Even her mother says*,*‘Why don’t you take her to a club?’ But sometimes my budget is a little tight*’ (Parent, 12).

### Theme 3: perceived benefits to weight management

This theme focuses on the perceived advantages and positive outcomes that parents observed from effectively managing their children’s weight. Parents noted improvements not only in the physical health of their children but also in their psychological well-being and family dynamics. These perceived benefits acted as motivators for parents to continue their efforts in managing their children’s obesity. This theme explores four perceived benefits of weight management as observed by parents, presented in the following sub-themes: improvements in physical health, psychological benefits and self-esteem, positive family dynamics, and satisfaction and sense of accomplishment.

#### Improvements in physical health

Parents consistently highlighted the physical benefits their children experienced after beginning weight management. Many noticed that their children had more energy, improved stamina, and better physical fitness. One parent shared, ‘*After my son lost weight*,*he became more active. He could play and run around without getting tired as quickly*,*and that was a huge relief for me.*’ (Parent, 6). Another parent observed, ‘*We started to see him enjoy sports again. He was more agile*,*and his overall health seemed to improve. It was like he was a different child*.’ (Parent, 9).

Parents also reported a reduction in obesity-related health issues, such as improved breathing, fewer complaints of fatigue, and better overall well-being. For example, one parent remarked, ‘*My daughter had issues with her breathing and felt exhausted all the time. Since starting on a healthier diet and regular exercise*,*she breathes better and isn’t as lethargic.*’ (Parent, 4).

#### Psychological benefits and self-esteem

Parents noted significant improvements in their children’s mental health and self-esteem as a result of weight management. The boost in self-confidence led children to engage more in social activities and school events, with parents observing greater happiness and sociability. For instance, one parent remarked, ‘*My child used to be so shy and withdrawn*,*but after losing weight*,*she has become more confident. She talks more and is happier to be around other kids*.’ (Parent, 2). These psychological benefits were closely tied to children’s positive perceptions of their own bodies and reduced experiences of bullying. For example, ‘*My son was bullied at school because of his weight. Since losing weight*,*he’s more comfortable with himself*,*and the bullying has stopped*.’ (Parent, 3).

#### Positive family dynamics and sense of accomplishment

Parents felt that the benefits of weight management extended beyond the individual child and positively influenced the entire family. As parents adopted healthier habits for their children, they also became more conscious of their own lifestyle choices. This led to more family-oriented activities, such as preparing healthy meals together and participating in physical activities as a family. One parent noted, ‘*The whole family got involved when we decided to improve our child’s weight. We started cooking healthier meals together*,*going for walks*,*and even playing sports in the park as a family*.’ (Parent, 8).

Additionally, parents found that as they engaged more in their children’s health, their overall relationship with their child improved. The process of supporting the child through weight management created stronger bonds and opened up more opportunities for open communication. For example, one parent shared, ‘*When I started supporting my daughter in her weight loss journey*,*we grew closer. It wasn’t just about her losing weight; it was about spending quality time together and working towards a goal*.’ (Parent, 10).

Parents reported a strong sense of personal satisfaction and accomplishment when they saw positive changes in their children, both physically and emotionally, as a result of weight management efforts. This sense of achievement reinforced their commitment to maintaining healthier lifestyle habits as a family. One parent noted, ‘*Seeing my child’s progress is very rewarding. It gives me hope that we’re doing the right thing*,*and it pushes us to keep going.*’ (Parent, 11). For many parents, the experience was not only about their child’s physical health but also about seeing them happier, more confident, and emotionally fulfilled, which validated the family’s collective efforts. As one parent shared, ‘*It is not just about my child being physically healthier*,*but also seeing him more confident*,*happier*,*and enjoying life. That makes all the effort worth it.*’ (Parent, 5).

### Theme 4: perceived motivators to addressing obesity

The findings for this theme reveal that parents are driven by various motivators to address childhood obesity in their children. Key motivators include concern for health risks, psychosocial well-being, and influence from role models and peers. These motivations stem from both immediate and long-term considerations and highlight the multifaceted nature of parents’ decision-making processes. This theme explores four key sub-themes that drive parents to address childhood obesity: concern for health risks, psychosocial well-being, influence of role models and peers, and family’s future aspirations.

#### Concern for health risks

The fear of long-term health complications emerged as a significant motivator for parents to manage their children’s weight. Parents were worried about conditions such as diabetes and cardiovascular disease, which they perceived as real threats to their child’s well-being. For instance, one mother explained, ‘*The doctor said my child was at risk for diabetes*,*and I was scared; I knew we had to change our lifestyle’* (Parent, 8). Another parent voiced a similar concern: ‘*I don’t want my son to suffer from illnesses like high blood pressure and cholesterol when he grows up; it’s better to start controlling his weight now*’ (Parent, 5). Such concerns about chronic diseases propelled parents to actively seek healthier lifestyles for their children.

Parents were also motivated by a desire to secure a healthier future for their children. They viewed addressing obesity as a proactive step to ensure their child would lead a happy and healthy life as they transitioned into adulthood. One parent shared, ‘*I want my daughter to grow up healthy*,*go to university*,*and not worry about her weight. If she learns healthy habits now*,*they will last for life*’ (Parent, 9). Another parent said, ‘*It’s not just about now*,*but about making sure my son can have a healthy future*,*without the risks that come with child and later adult obesity’* (Parent, 2). As one parent reflected, *‘The idea that my son could develop serious conditions like diabetes or heart disease in the future terrifies me. This fear motivates me to work on his weight now’ (Parent*,*7).*

#### Psychosocial well-being

This sub-theme encompasses parents’ concerns for their children’s mental health, self-esteem, and desire for social acceptance. Parents observed that weight management positively impacted their children’s confidence and social interactions, motivating them to address obesity. One father shared, ‘*My daughter used to feel very shy and embarrassed in social settings because of her weight. Seeing her cry made me realise that we needed to help her lose weight’* (Parent, 4). Another parent expressed a similar concern, ‘*I don’t want my child to feel different or excluded because of his weight. It affects his happiness and confidence’* (Parent, 10).

In addition to emotional well-being, parents were motivated by a desire to protect their children from bullying and social exclusion. One mother explained, ‘*My son was bullied at school for being overweight; it broke my heart. I want him to fit in with his friends and not be teased anymore’* (Parent, 3). Another parent shared how social exclusion impacted their child’s happiness, ‘*It’s tough to see your child left out of games because they’re not as fit as other kids. I wanted to give him a chance to be included’* (Parent, 11). Overall, the combined concerns for both psychological well-being and social acceptance underscore the significance of psychosocial motivators in parental decisions to support their children’s weight management journey.

#### Influence of role models and peers

Positive role models within the community or family served as catalysts for parents to take action in managing their children’s weight. Witnessing the success stories of others provided motivation and inspiration. As one parent reflected, ‘*I saw a child in our neighbourhood who lost a lot of weight. I thought*,*if he could do it*,*so can we’* (Parent, 6). Another parent shared how a relative’s weight-loss journey influenced their decision: ‘*My sister’s son managed to lose weight*,*and seeing that transformation made me believe we could do the same for our child’* (Parent, 7). These experiences among peers and family members inspired parents to take action.

These findings align with the constructs of the Health Belief Model (HBM), as detailed in Table 3, which summarises how each theme reflects key elements of the HBM and contributes to understanding parents’ motivations and barriers to addressing childhood obesity in Riyadh, Saudi Arabia. The discussion section next offers a detailed examination of our findings, including how each theme relates to the constructs of the HBM and aligns with existing literature.

**Table 3:** Alignment of study themes with HBM constructs

Theme	HBM construct	Explanation
**Theme 1**: Overall perceptions towards childhood obesity	Perceived susceptibility and severity	Cultural and societal norms initially obscure the health risks of childhood obesity; however, as awareness of health implications increases, parents start perceiving obesity as a serious threat to their child’s health.
**Theme 2**: Perceived barriers to weight management	Perceived barriers	Barriers such as cultural beliefs, limited time, lack of institutional support, and financial constraints impede parents’ efforts to manage their children’s weight effectively.
**Theme 3**: Perceived benefits to weight management	Perceived benefits	Observing improvements in physical health, psychological well-being, and family relationships encourages parents to maintain healthy lifestyle changes for their children.
**Theme 4**: Perceived motivators to addressing obesity	Cues to action and health motivation	Concerns about health risks, social influences from role models, and aspirations for a healthier future prompt parents to initiate and sustain efforts to manage their children’s weight.

## Discussion

The findings of this study provide important insights into Saudi parents’ perceptions of childhood obesity and its management. Four main themes emerged from the analysis: overall perceptions towards childhood obesity, perceived barriers to weight management, perceived benefits to weight management, and perceived motivators to weight management. These themes highlight the complex interplay of cultural beliefs, perceived challenges, observed benefits, and motivating factors that influence parental approaches to childhood obesity. The results align with previous research, yet they also contribute new perspectives, particularly regarding the role of parental perceptions in the management of childhood obesity in Riyadh, Saudi Arabia. This discussion addresses each theme in relation to the relevant literature, exploring the implications for public health strategies and family-centred interventions that could be tailored to the specific needs of the Saudi context. Additionally, the discussion will link these findings to the HBM, illustrating how the model’s components—such as perceived severity, susceptibility, and barriers—can contextualise parents’ shifting views on childhood obesity and highlight potential intervention strategies.

### Overall perceptions towards childhood obesity

The findings of this study reveal that many parents in Riyadh initially perceived childhood obesity as a sign of good health, rooted in cultural beliefs that associate a “chubby” child with vitality, wealth, and beauty. This perception aligns with previous research showing that, historically, in the Gulf States—including Bahrain, Kuwait, Oman, Qatar, Saudi Arabia, and the United Arab Emirates—as well as other Arab, African, and European countries with similar cultural and religious values, childhood obesity is often viewed positively [28, 29]. These cultural and societal perceptions have, in many cases, delayed the recognition of obesity as a health issue, as reflected by the experiences of parents who participated in this study. Similar findings were reported in another study completed in Riyadh that parental misperceptions surrounding childhood obesity present a barrier to recognising obesity as a health risk [30]. Addressing these cultural misperceptions is essential to promote early recognition and intervention in childhood obesity management in Riyadh.

However, parents’ perspectives from our study began to shift as they observed the physical and social challenges their children faced, such as difficulty performing physical activities, breathing issues, and social stigma. This change in perception mirrors findings from other studies in Saudi Arabia, which document a growing awareness of the health risks and social difficulties associated with childhood obesity over the past few decades [7, 31]. Yet, some parents in Riyadh may still struggle to recognise the urgency of addressing obesity, as the deep-seated perception of obesity as a positive trait can hinder efforts to promote healthy weight management.

The findings from Theme 1 align with the HBM, which suggests that as individuals become more aware of the severity and susceptibility to health risks, they are more likely to take preventive action [32]. For many parents in this study, a growing awareness of the health risks associated with childhood obesity served as a turning point, prompting them to seek interventions and actively manage their children’s weight. This dynamic shift in perceptions echoes findings in other cultural contexts where childhood obesity is initially seen as a sign of good health. In such settings, it often takes tangible health complications for parents to reassess and revise their beliefs about childhood obesity [33]. The HBM helps contextualise this evolution in parental beliefs by illustrating how cultural and social norms can act as perceived barriers, hindering the recognition of childhood obesity as a serious health risk. The findings highlight the importance of addressing these cultural beliefs early on through health education, as well as the need for interventions that raise awareness about the long-term health implications of obesity. This approach can help shift perceptions, encouraging preventive action and healthier behaviours for effective childhood obesity management in Riyadh.

### Perceived barriers to managing obesity

The findings of this study reveal several key barriers that parents in Riyadh face in managing childhood obesity. These barriers reflect cultural, social, and practical challenges that complicate parents’ efforts to promote healthier lifestyles for their children. This theme explores four key subthemes: cultural and social barriers, dietary habits due to lack of time, lack of support, and financial barriers.

A significant barrier identified was the influence of cultural and social norms, which reinforced the idea that a heavier child is healthier. This finding is consistent with research showing that cultural beliefs significantly impact how parents perceive and respond to childhood obesity [34]. The parents in this study often found it challenging to address obesity due to societal reinforcement of chubbiness as a positive trait, making it harder to prioritise interventions for their children’s health.

Busy work schedules and the availability of fast food were additional barriers noted by parents participated in our study. Many parents reported that time constraints limited their ability to prepare nutritious meals, leading to reliance on calorie-dense, processed foods for convenience. Time constraints often drive families towards convenient, but unhealthy, food options [35]. This trend is particularly evident in urbanising regions like Saudi Arabia, where rapid development has made processed and fast foods more accessible and attractive [13]. In line with findings from other studies, one parent explained that their prolonged working hours often prevented them from preparing balanced meals at home, resulting in more frequent fast-food consumption for their children.

Parents expressed frustration at the lack of consistent support from schools, healthcare providers, and family members in managing their children’s obesity. Many felt that schools could do more to promote healthy eating and physical activity, such as providing healthier food options in canteens and engaging parents about their children’s health. Similar findings reported in another study where parents highlighted the absence of clear guidance from healthcare providers, which left them feeling unsupported in managing their children’s weight [36]. Some parents also mentioned that family gatherings often undermined their efforts by encouraging unhealthy eating habits, adding to the challenge of creating a supportive environment for their children’s weight management.

Financial constraints significantly impacted some parents’ ability to prioritise healthier food options and physical activities in Riyadh. Healthier foods were often perceived as more expensive and less accessible than processed alternatives, making it challenging to maintain a nutritious diet on a budget. One parent shared that, while they wanted to enrol their child in a sports club, the cost was prohibitive, illustrating how financial barriers restrict opportunities for regular physical activity. These constraints highlight the role of economic factors in shaping parents’ ability to promote healthier behaviours for their children. These findings align with research conducted in Jeddah, Saudi Arabia, which indicates that financial limitations can hinder access to healthy foods and physical activity opportunities, thereby contributing to childhood obesity [37]. Similar findings reported in another study in Riyadh, Saudi Arabia, show that unemployed fathers and low-income families are at higher risk of having overweight or obese children [7].

The findings from Theme 2 align with the HBM, which suggests that individuals are more likely to engage in health-promoting behaviours when they perceive a serious health threat, recognise barriers to change, and feel supported in overcoming these barriers [38]. According to the HBM and our study findings, cultural and social influences, time constraints affecting dietary habits, insufficient support, and financial limitations can significantly hinder behaviour change by diminishing the perceived severity of childhood obesity, thus reducing motivation for action. In line with the HBM, addressing these barriers by providing targeted health education and institutional support could increase parents’ perceived efficacy in managing their children’s weight, leading to greater engagement with weight management practices in Riyadh.

### Perceived benefits to weight management

This theme explores the perceived benefits that parents observed from effectively managing their children’s weight, covering improvements in physical health, psychological well-being, and positive family dynamics. These perceived benefits reflect the interconnected impacts of physical and mental health improvements and underscore the role of family involvement in maintaining children weight management practices by parents in Riyadh.

In terms of physical health, parents noted significant improvements in their children’s energy levels, stamina, and overall fitness after implementing weight management practices. Many reported that their children became more active and could engage in physical activities without easily tiring, which provided relief and reassurance. These findings are consistent with broader literature showing that weight management can lead to better cardiovascular health and higher physical activity levels, contributing to an overall improved quality of life for children [39, 40]. Parents found these physical health gains encouraging, reinforcing their efforts and determination to continue supporting healthier lifestyle habits for their children.

Psychologically, parents observed notable enhancements in their children’s confidence, social engagement, and self-esteem as a result of weight management. For instance, as children experienced physical health benefits, they also appeared more willing to participate in social activities and school events, which positively impacted their happiness and sociability. Parents described how these psychological improvements were closely linked to children’s positive body image and reduced bullying experiences. This dual benefit of physical and mental well-being aligns with findings from similar studies, highlighting that improvements in self-esteem and social interactions are often pivotal motivators for parents to persist in managing their children’s weight. For instance, study found that obese children in Western Saudi Arabia exhibited higher rates of emotional and social problems compared to their normal-weight peers, indicating that addressing these issues can be a significant factor in weight management efforts [41].

Finally, weight management positively influenced family dynamics, strengthening bonds and creating a sense of shared accomplishment. Many parents reported that the entire family became more involved in healthy lifestyle changes, participating in activities such as cooking nutritious meals and engaging in family-oriented physical activities. Our findings were similar to other studies completed in Saudi Arabia. For instance, a study found that parental engagement in their children’s dietary habits and physical activities led to improved family cohesion and collective participation in health-promoting behaviours [42–44]. This shared commitment fostered a supportive family environment, further reinforcing healthy habits. Parents also expressed a sense of personal satisfaction and pride in seeing the positive changes in their children, which validated their collective efforts.

The findings from this theme align with the HBM, which suggests that individuals are more likely to engage in health-promoting behaviours when they perceive clear benefits and feel supported in making changes [38]. In our study, the recognition of substantial benefits—such as improved physical health, psychological well-being, and strengthened family bonds—motivated parents to sustain weight management efforts. These insights from our study in Riyadh suggest that interventions which emphasise both immediate and long-term benefits of weight management for children can be particularly effective in strengthening parental motivation. By highlighting these benefits within the framework of the HBM, parents may be more consistently engaged in supporting healthier lifestyle practices for their children.

### Perceived motivators to addressing obesity

Parents in Riyadh are motivated by multiple factors to address childhood obesity in their children, with key motivators including concerns about health risks, the desire to improve psychosocial well-being, the influence of role models and peers, and aspirations for a healthier future. These motivators reveal a mix of immediate and long-term considerations that guide parents’ commitment to managing their children’s weight. The following sections discuss each of these motivators, highlighting how they shape parents’ ongoing engagement in promoting a healthier lifestyle for their children.

In contrast to the initial perceptions of health risks discussed earlier, where obesity was not always viewed as a serious threat, this theme highlights the ways in which growing awareness of specific health risks serves as a key motivator for parents to address their children’s weight. Concern for health risks emerged as the primary motivator, with parents worried about long-term conditions like diabetes and cardiovascular disease, commonly associated with childhood obesity [2]. Similar studies from Saudi Arabia indicate that parental awareness of obesity-related health risks significantly encourages preventive actions, as many parents aim to protect their children from severe health complications [7, 45]. As parents in this study became more aware of these potential health issues, their motivation to make lifestyle changes—such as healthier diets and increased physical activity—strengthened.

Psychosocial well-being was also a notable motivator, as parents were driven by a desire to improve their children’s mental health and social acceptance. Many parents observed that their children faced social exclusion or bullying due to their weight, leading to decreased self-esteem and feelings of isolation. In response, parents became more committed to managing obesity to promote their children’s confidence and social interactions. This finding is supported by previous research in Saudi Arabia, where concerns for children’s social and psychological experiences play a substantial role in motivating parents to address weight issues [3]. These insights suggest that interventions addressing both physical and psychological aspects of obesity, such as comprehensive health education and mental health support, may be particularly effective for families in Riyadh.

Influence from role models and peers further motivated parents, as observing successful weight management in their community reinforced their belief in the possibility of positive outcomes for their children. When parents saw others achieve weight loss goals, they felt empowered to undertake similar efforts, highlighting the role of social support in motivating health-related behaviour. Research in Saudi Arabia confirms that social influences, especially in close-knit communities, play a significant role in shaping health behaviours, as individuals often draw inspiration and motivation from their immediate social circles [46, 47].

Lastly, aspirations for their children’s future served as a strong motivator, with parents viewing obesity management as essential to ensuring a long, healthy life. Parents expressed the desire to establish lasting healthy habits in their children, seeing weight management as a way to safeguard their children’s future happiness and success. This aligns with findings from other studies in Saudi Arabia, where long-term health considerations are increasingly recognised by parents as vital for their children’s well-being. However, a study from Al-Qassim, Saudi Arabia, reveals that many parents misjudge their children’s weight status, with a large proportion of parents of overweight and obese children perceiving them as having a normal weight, underscoring a prevalent gap in parental awareness that may hinder timely obesity intervention efforts [15].

In line with the HBM, these findings suggest that as parents in Riyadh perceive the significant health benefits of weight management for their children, their motivation to promote healthier behaviours increases. The HBM posits that when health threats like obesity are clearly understood, and when there is community and peer support, parents are more likely to engage actively in health-promoting practices [48]. Interventions that emphasise both immediate and future health benefits may enhance parental motivation, making it easier for parents to sustain these lifestyle changes for the long term.

### Strengths and limitations of study

This study’s qualitative approach, utilising an interpretive paradigm, provides rich insights into the experiences of Saudi parents, particularly through the hybrid thematic analysis that balanced both pre-identified and emerging themes. This depth of understanding provides an important context to the issue of childhood obesity in Riyadh, offering culturally grounded insights that may not be captured by quantitative methods. Semistructured interviews allowed for a deep understanding of cultural and social factors influencing childhood obesity, with data saturation indicating a robust sample. The study also reached data saturation by the tenth interview, indicating that a sufficient number of participants were interviewed to uncover meaningful patterns and themes.

However, a key limitation is its focus on a relatively small and specific sample, consisting of only twelve parents from Riyadh, which restricts the generalisability of the findings to all Saudi parents. While this sample size is appropriate for qualitative research, the findings may not be representative of all Saudi parents or those from different regions or socioeconomic backgrounds. Moreover, the study primarily included participants who were already actively involved in discussions about obesity, which may have introduced selection bias. As a result, the experiences of parents less engaged or informed about obesity may not have been adequately captured.

Additionally, since the study focused on parents of children who were either overweight or obese, there may be differences in how parents perceive and manage obesity depending on the severity of their child’s condition. Parents of children classified as obese may have more urgency or a heightened awareness of the need for intervention compared to parents of children who are simply overweight. This distinction may have influenced the results, and the findings may not fully capture the experiences of parents with overweight children who have not yet reached the threshold of obesity. Further research could explore the differences in perceptions and strategies between parents of overweight and obese children to deepen understanding in this area. Another limitation of this study is the predominance of female participants, which may have influenced the findings, as mothers typically play a central role in caregiving and health-related decision-making in Saudi Arabia. Including fathers in future research could provide a broader perspective on parental perceptions of childhood obesity in Saudi Arabia.

Furthermore, since the interviews were conducted in Arabic and then translated into English, there is a potential for nuances and meanings to have been lost in translation, despite efforts to ensure accuracy through back-translation and member checking. Despite these limitations, this research offers valuable context-specific insights and lays the groundwork for further exploration into the cultural influences on childhood obesity management in Riyadh, Saudi Arabia.

## Conclusion

Understanding Saudi parents’ perceptions of childhood obesity is fundamental to developing effective interventions that resonate with cultural norms and social realities. The findings of this study highlight how cultural beliefs, social practices, and economic challenges significantly influence parents’ views on childhood obesity and their capacity to manage it. Addressing these perceptions is essential for any meaningful public health strategy. The results underscore the importance of culturally sensitive interventions that consider parents’ motivations, perceived barriers, and evolving understanding of obesity. To enhance the success of obesity management and prevention, healthcare providers and policymakers must align their efforts with the lived experiences of families, thus promoting improved health outcomes for Saudi children.

## Electronic supplementary material

Below is the link to the electronic supplementary material.

Supplementary Material 1

## Data Availability

The authors confirm that the data supporting the findings of this study are available within the article. The data supporting this study’s findings are also available from the corresponding author upon reasonable request.

## Abbreviations

BMI: Body Mass Index
HBM: Health Belief Model
