# Supplementary material for: Exploring parents’ perceptions and experiences of childhood obesity and management in Riyadh, Saudi Arabia: an interpretive qualitative study
Source: BMC Public Health. 2024 Dec 18;24:3452. doi: 10.1186/s12889-024-21014-6 (PMC11657218; doi:10.1186/s12889-024-21014-6)
Supplement: Supplementary file 1 — Supplementary Material 1 [file 12889_2024_21014_MOESM1_ESM.docx]

**Supplementary Materials**

**Semi-structured Interview Guide**

**Demographics:**

1. Please state your name: ……….
2. Are you the child's mother or father? ………..
3. Are parents living with each other?
4. What is your age? ………..
5. What is your educational level? ………..
6. What is your occupation? ………..
7. Age of your child? ………..
8. Child's gender……
9. What is your child’s height and weight? [Or BMI if known]? ………..

**Questions:**

1. Could you please tell me about your experience with your child's obesity? (General Question).
2. How did you know that your child was overweight or obese?
3. How do you feel your child became overweight or obese? if they think that their child's body is not obese, why do they believe that?
4. Talk about your thoughts on your child's weight and perceptions of unhealthy and a healthy child's weight.
5. How can a child's general health be impacted by childhood obesity? (How do you think about obesity that is out of control?).
6. How do you think it would help your child if she or he lost some weight? (what difference would it make to his life?)
7. Has your child ever tried or participated in any weight management programs with your child? How did they go? If unsuccessful, why was that? (What obstacles excited?)
8. What encourages you to control your child's weight and engage your child in weight management?
